# Supplementary material for: The AT-hook is an evolutionarily conserved auto-regulatory domain of SWI/SNF required for cell lineage priming
Source: Nat Commun. 2023 Aug 4;14:4682. doi: 10.1038/s41467-023-40386-8 (PMC10403523; doi:10.1038/s41467-023-40386-8)
Supplement: Supplementary file 1 — Supplementary Information [file 41467_2023_40386_MOESM1_ESM.pdf]

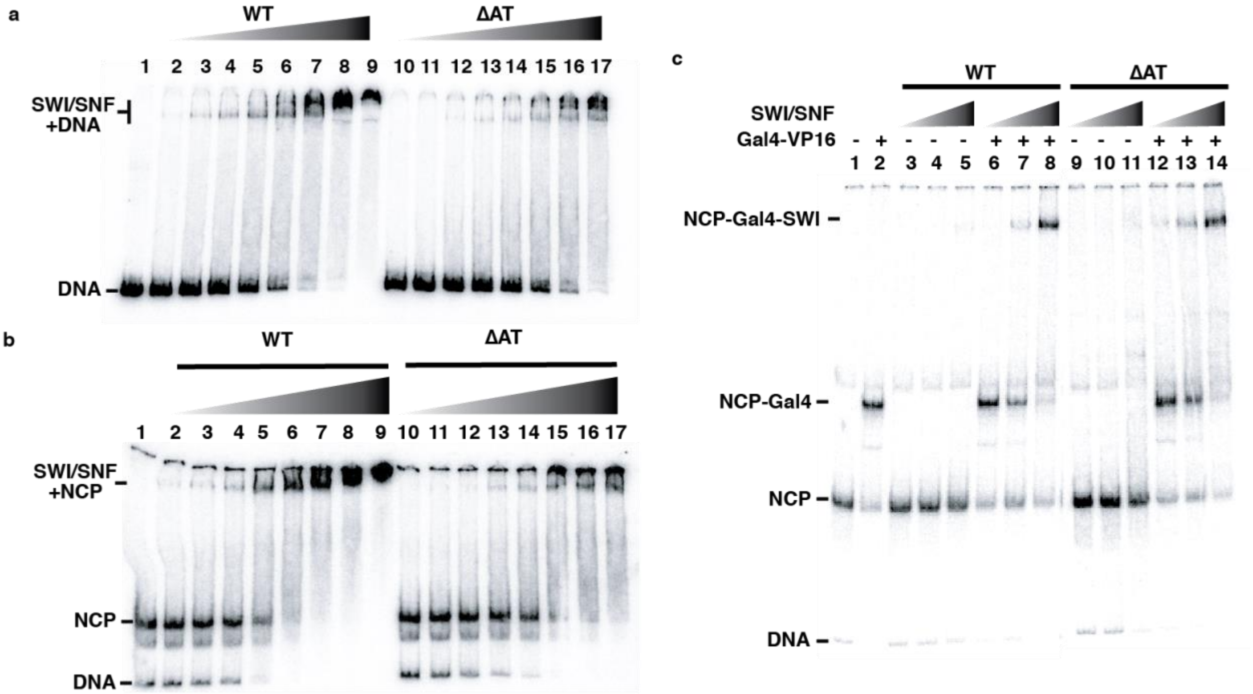

Supplementary Fig. 1: The AT-hooks of Snf2 are not required for recruitment of yeast SWI/SNF.

(a-b) Representative native gel images used in Figures 3a and S3b to measure (a) the relative affinity of WT and ΔAT SWI/SNF for nucleosomes and (b) competition for WT and ΔAT SWI/SNF binding between free DNA and nucleosomes. **(c)** Recruitment of WT and ΔAT SWI/SNF to nucleosomes, as shown by EMSA, is mediated by the transcription factor Gal4-VP16. SWI/SNF under these conditions in which competitor DNA is present cannot be recruited to nucleosomes without Gal4-VP16 bound to the extranucleosomal DNA (compare lanes 3-5 to 6-8 for WT and lanes 9-11 to 12-14 for ΔAT SWI/SNF). This image is representative of four independent replicates.

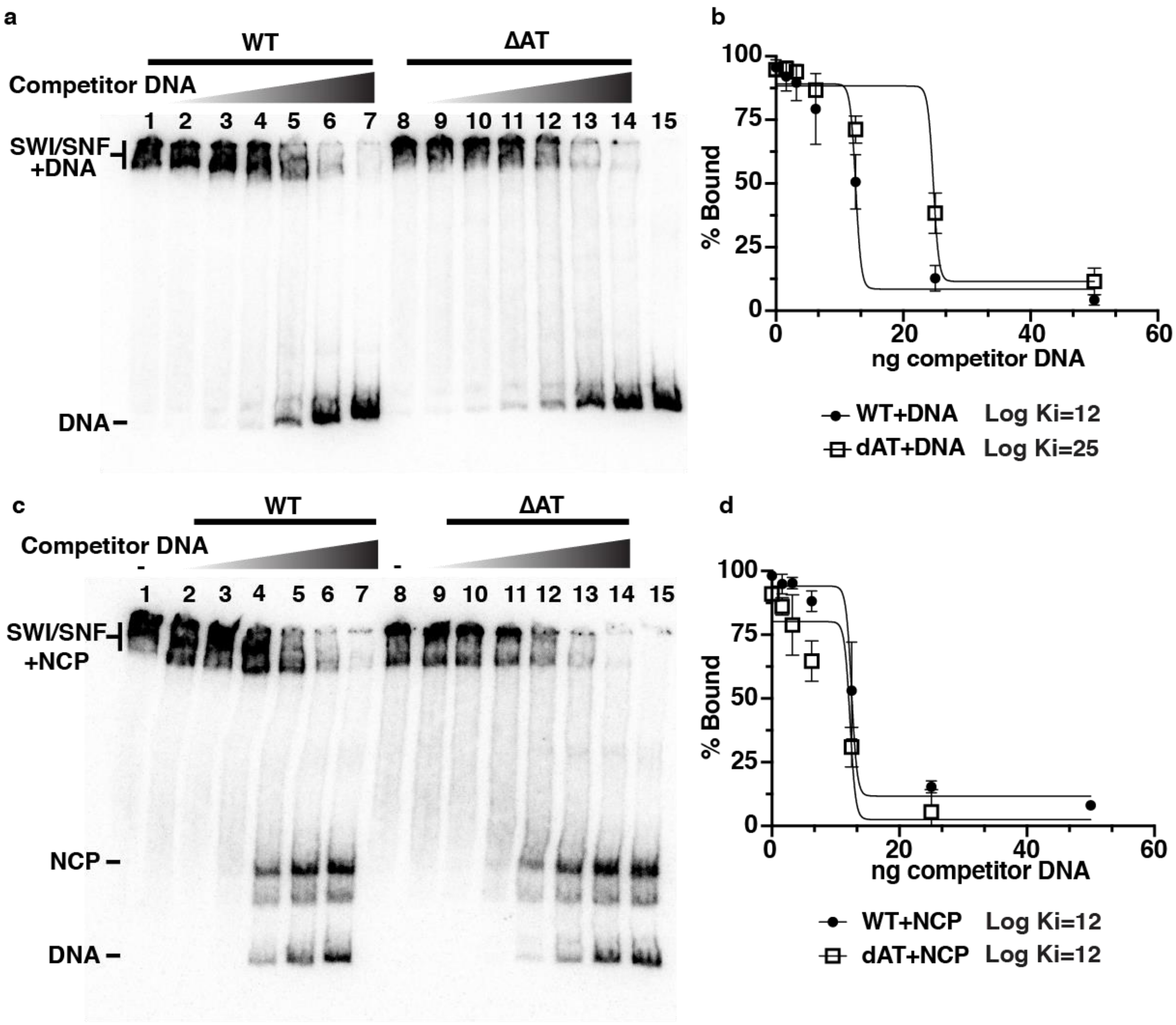

Supplementary Fig. 2: Competition of WT and ΔAT SWI/SNF binding to DNA or nucleosomes.

(a,c) Binding of WT or ΔAT SWI/SNF to either (a) free DNA or (c) nucleosomes was competed with unlabeled pUC19 plasmid DNA and tracked by EMSA. **(b,d)** A total of 4 replicates were performed for each and the percent bound versus SWI/SNF concentration is plotted for the mean value with error bars showing the standard deviation. Source data are provided as a Source Data File.

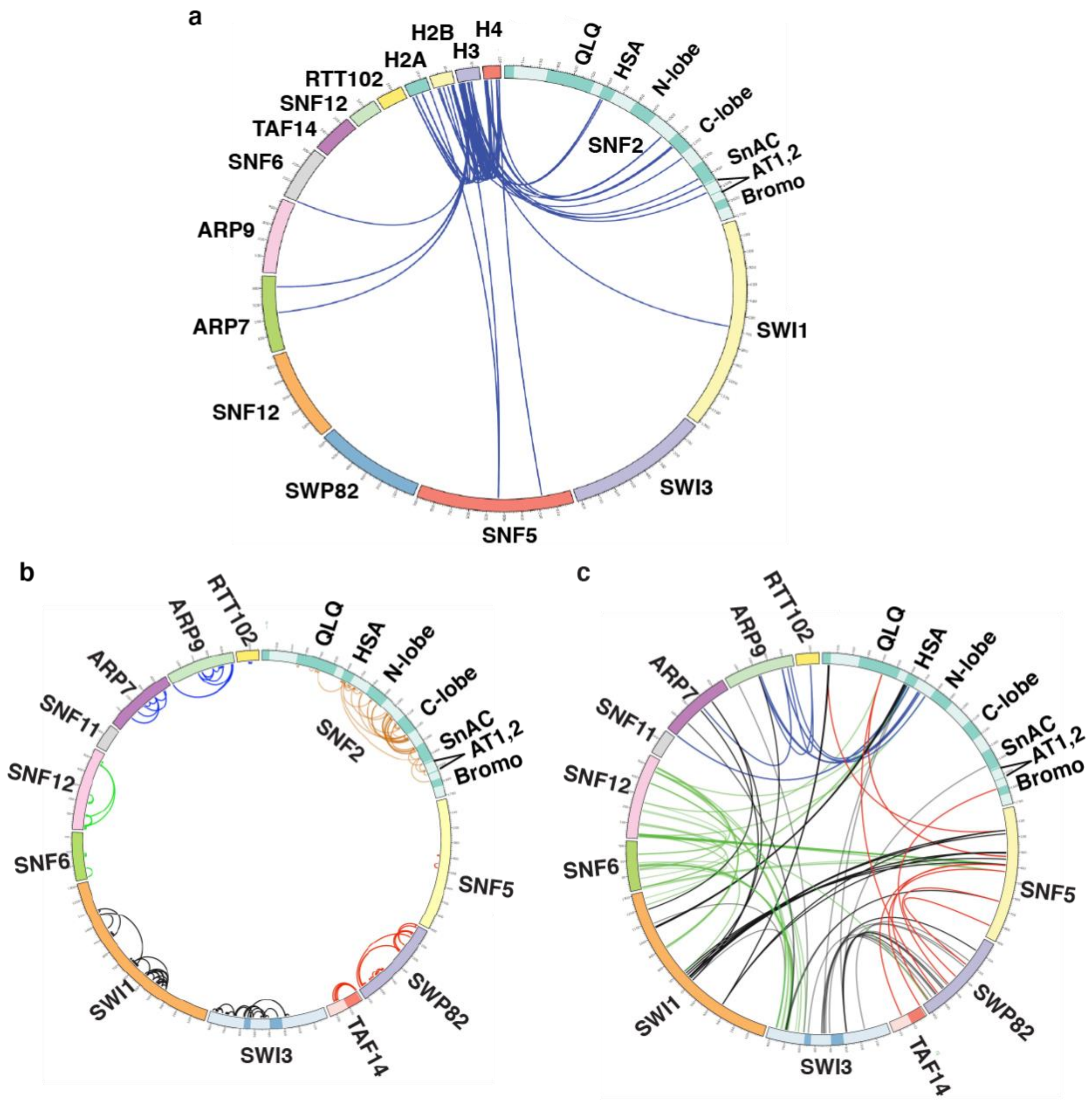

Supplementary Fig. 3: AT-hook switches binding partner(s) upon SWI/SNF binding nucleosomes

(a) The Lys-Lys crosslinking patterns between histones and the SWI/SNF subunits are shown for the nucleosome-bound SWI/SNF complex. **(b-c)** The SWI/SNF crosslinking pattern is depicted in a Circos format for (b) intra- and **(c)** inter-subunit crosslinking for WT SWI/SNF. Different colored lines are used to group particular subunits that form submodules within the SWI/SNF complex.

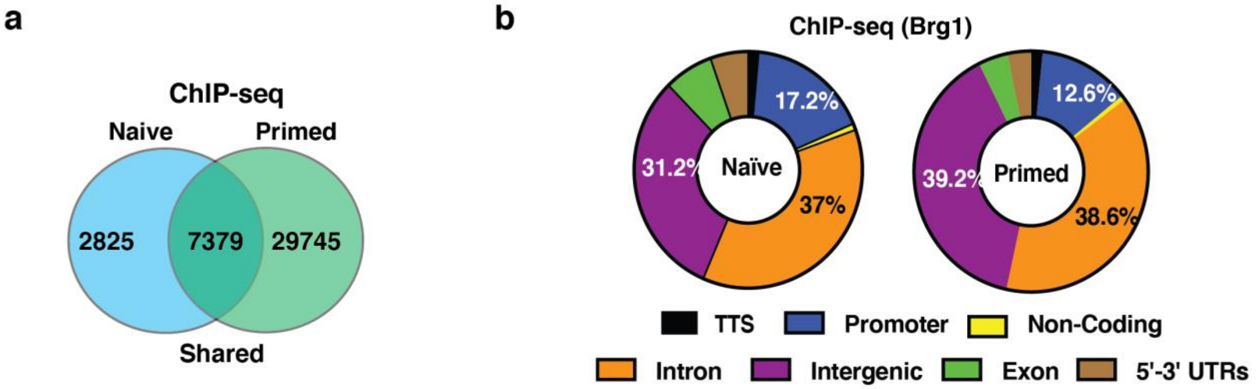

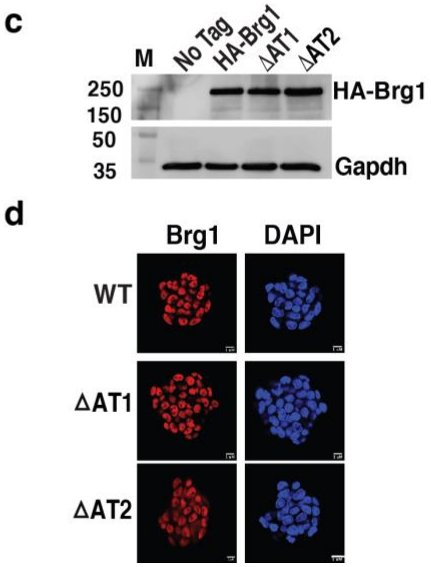

e

Normalized Average Enrichment of esBAF and gBAF subunits

	WT-Naïve	dAT-Naïve	WT-Primed	dAT-Primed
**Brg1**	4.72 ± 0.00	4.72 ± 0.00	4.72 ± 0.00	4.72 ± 0.00
**Arid1a**	3.78 ± 0.13	3.98 ± 0.13	2.03 ± 0.73	1.10 ± 0.10
**Smarcc1**	3.45 ± 0.12	3.33 ± 0.50	2.65 ± 0.24	2.03 ± 0.59
**Smarcd1**	3.52 ± 0.08	3.88 ± 0.25	2.76 ± 0.11	1.62 ± 0.18
**Smarcd2**	4.00 ± 0.08	4.30 ± 0.43	3.05 ± 0.39	1.87 ± 0.04
**Actl6a**	4.98 ± 0.12	5.79 ± 0.14	4.83 ± 1.46	3.92 ± 0.22
**Snf5**	5.13 ± 0.16	5.88 ± 0.35	4.11 ± 0.52	3.32 ± 0.15
**Dpf2**	4.44 ± 0.04	4.89 ± 0.19	3.16 ± 0.44	2.24 ± 0.13
**Brd9**	4.76 ± 0.42	4.47 ± 0.51	3.61 ± 0.48	2.82 ± 0.63
**Gltscr1L**	4.12 ± 0.06	3.92 ± 1.23	2.98 ± 0.54	1.72 ± 0.01
**GltscrR1**	4.40 ± 0.94	4.36 ± 2.30	2.41 ± 0.40	1.02 ± 0.49
**Brd7**	5.12 ± 0.08	6.22 ± 0.10	3.04 ± 0.12	2.44 ± 0.10
**Phf10**	4.56 ± 0.45	5.24 ± 0.95	2.09 ± 0.62	1.72 ± 0.55

Supplementary Fig. 4: The AT-hook deletion does not affect Brg1 expression and SWI/SNF complex integrity. (**a**) Venn diagrams show the number of Brg1 peaks detected in naïve and primed cells using ChIP-seq. **(b)** Pie charts show the genome-wide distribution of Brg1 using ChIP-seq. **(c)** Western blot for HA-tagged Brg1 expression in WT and dAT mutants mESCs in naïve condition; untagged cells were used as a control, and Gapdh was used as a loading control. The standard protein markers and their molecular weight in kDa are shown in M. **(d)** The localization and expression of Brg1 in WT and dAT mutants is shown by immunofluorescence in naïve cells**. (e)** The table shows the average enrichment of es-BAF and gBAF components in WT and AT-hook deleted mutant cells detected by mass spectrometry in both naïve and primed states. The numbers indicate the normalized average enrichment of peptides of respective proteins and ± represents the standard error of two replicates. Enrichment factor normalization was done using Brg1. Source data are provided as a Source Data File.

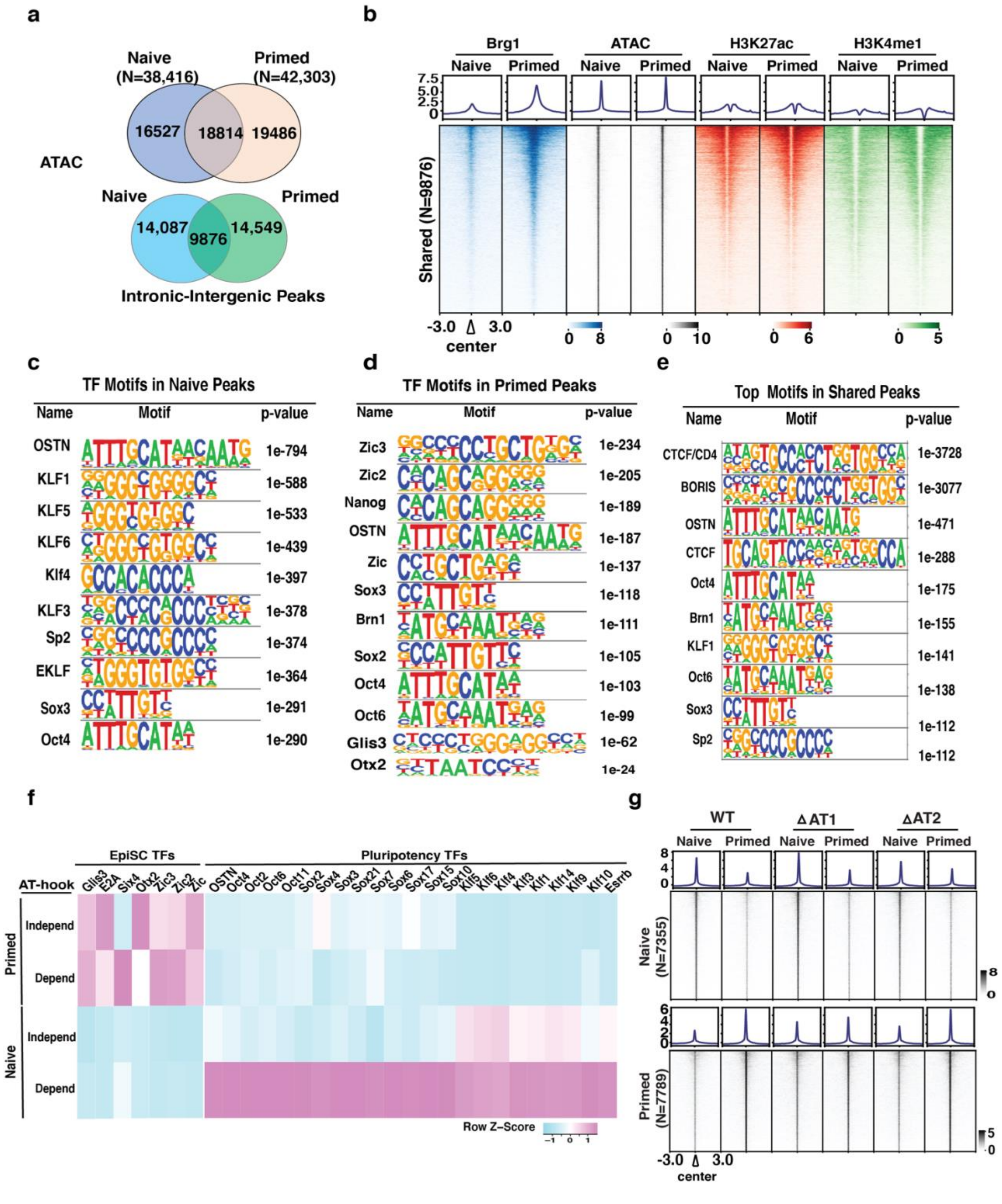

Supplementary Fig. 5: Loss of AT-hook alters accessibility at intronic-intergenic sites. **(a)** Venn diagrams show cell-type specific and shared ATAC-seq peaks (upper panel) and those only in intergenic and intronic regions (lower panel) in WT and dAT mutants in naïve and primed cells. (**b**) Brg1 localization (blue), ATAC-signal (grey), and active enhancer histone marks

(H3K27ac [red], and H3K4me1 [green]) at shared intronic-intergenic ATAC-seq peaks (TF-dependent). Signals are sorted based on high to low. N represents the total number of ATAC-seq peaks. **(c-e)** Table showing the top transcription factors motif enriched in naïve (c), primed (d), and shared (e) ATAC-seq intronic-intergenic peaks using HOMER. **(f)** Heatmap showing the pluripotency and epiblast specific (EpiSC) specific transcription factors (TFs) motif enrichment between AT-hook dependent -vs- AT-hook independent ATAC-seq intronic-intergenic peaks in naïve and primed states. **(g)** Heatmaps showing ATAC signals in the two independent clones of the AT-hook mutant in naïve (top) and primed (bottom) states versus WT. ATAC signals are sorted based on WT.

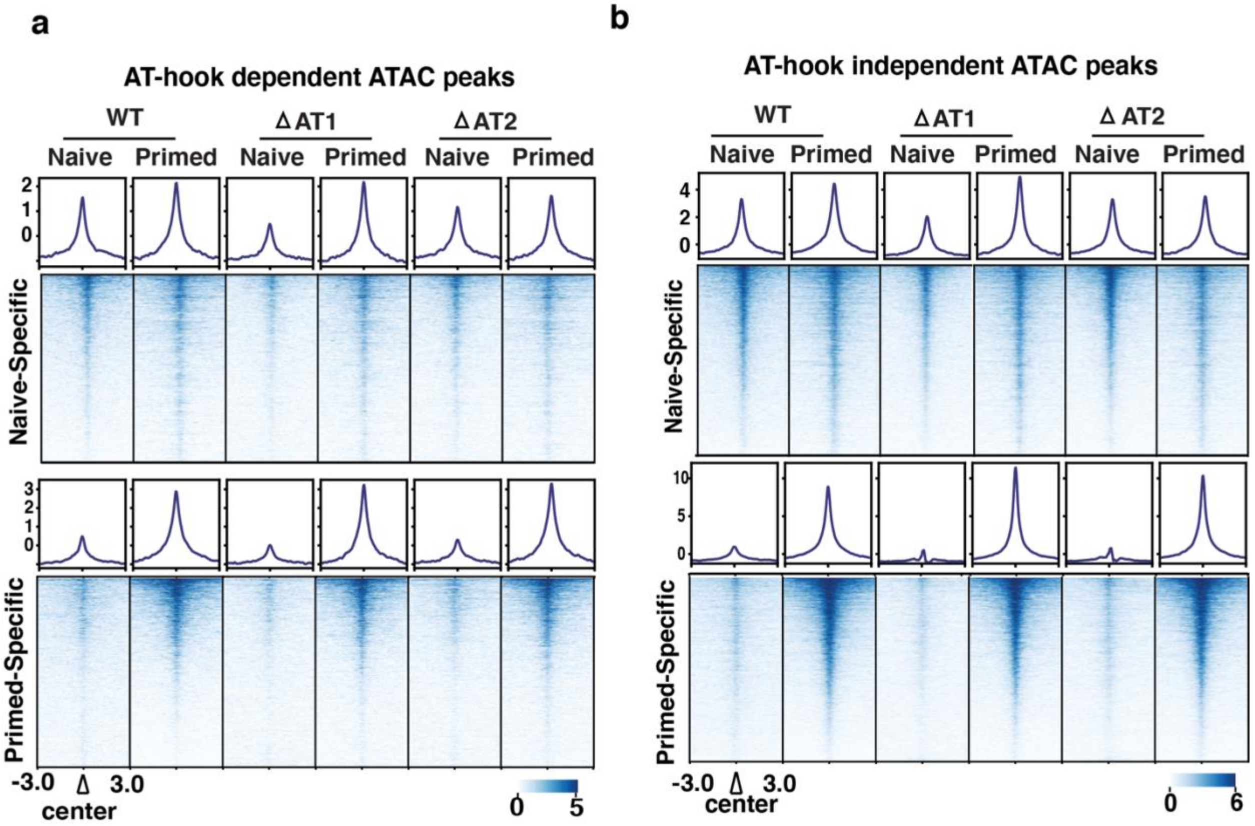

Supplementary Fig. 6: Heatmaps showing Brg1 localization at AT-dependent (a) and AT-independent (b) ATAC intronic-intergenic sites. ChIP signals are sorted based on WT in each group.

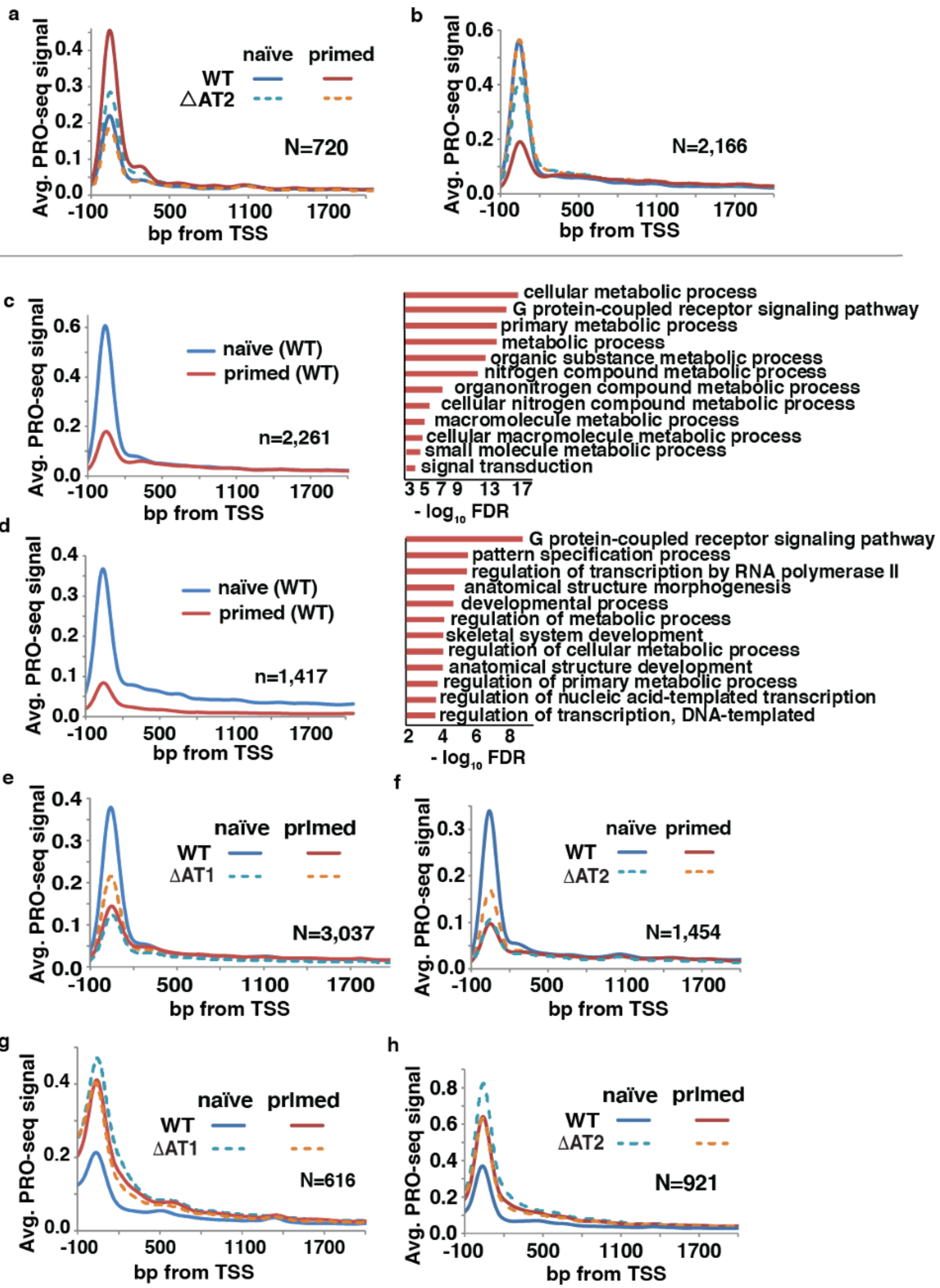
a
Avg. PRO-seq signal
bp from TSS
naïve primed
WT
△AT2
N=720
b
N=2,166
c
naïve (WT)
primed (WT)
n=2,261
cellular metabolic process
G protein-coupled receptor signaling pathway
primary metabolic process
metabolic process
organic substance metabolic process
nitrogen compound metabolic process
organonitrogen compound metabolic process
cellular nitrogen compound metabolic process
macromolecule metabolic process
cellular macromolecule metabolic process
small molecule metabolic process
signal transduction
- log10 FDR
d
n=1,417
G protein-coupled receptor signaling pathway
pattern specification process
regulation of transcription by RNA polymerase II
anatomical structure morphogenesis
developmental process
regulation of metabolic process
skeletal system development
regulation of cellular metabolic process
anatomical structure development
regulation of primary metabolic process
regulation of nucleic acid-templated transcription
regulation of transcription, DNA-templated
e
ΔAT1
N=3,037
f
ΔAT2
N=1,454
g
N=616
h
N=921

Supplementary Fig. 7: Transcriptional defects due to the loss of the AT-hook. (a-b) PRO-seq metagene analysis (upstream TSS -100 bp to +300 bp downstream of TSS) for (a) WT and (b) ΔAT2 mutant Brg1 is shown that is similar to Figures 7d-e for ΔAT1. **(c-d)** PRO-seq metagene analysis for genes upregulated in the naïve stage for WT cells is shown on the left. The genes in (c) are those in which only promoter-proximal paused RNAPII is detected, whereas in (d) RNAPII is also transcribing through the gene body. On the right is the corresponding bar graphs for the gene ontology analysis. (**e-h**) PRO-seq metagene analysis is shown for genes dysregulated in (e,g) ΔAT1 and (f,h) ΔAT2 for the naive stage. In (e-f) these genes fail to be activated in the naïve stage when the AT-hook is deleted and in (g-h) these genes are activated upon loss of the AT-hook when they are normally inactive in the naïve stage.

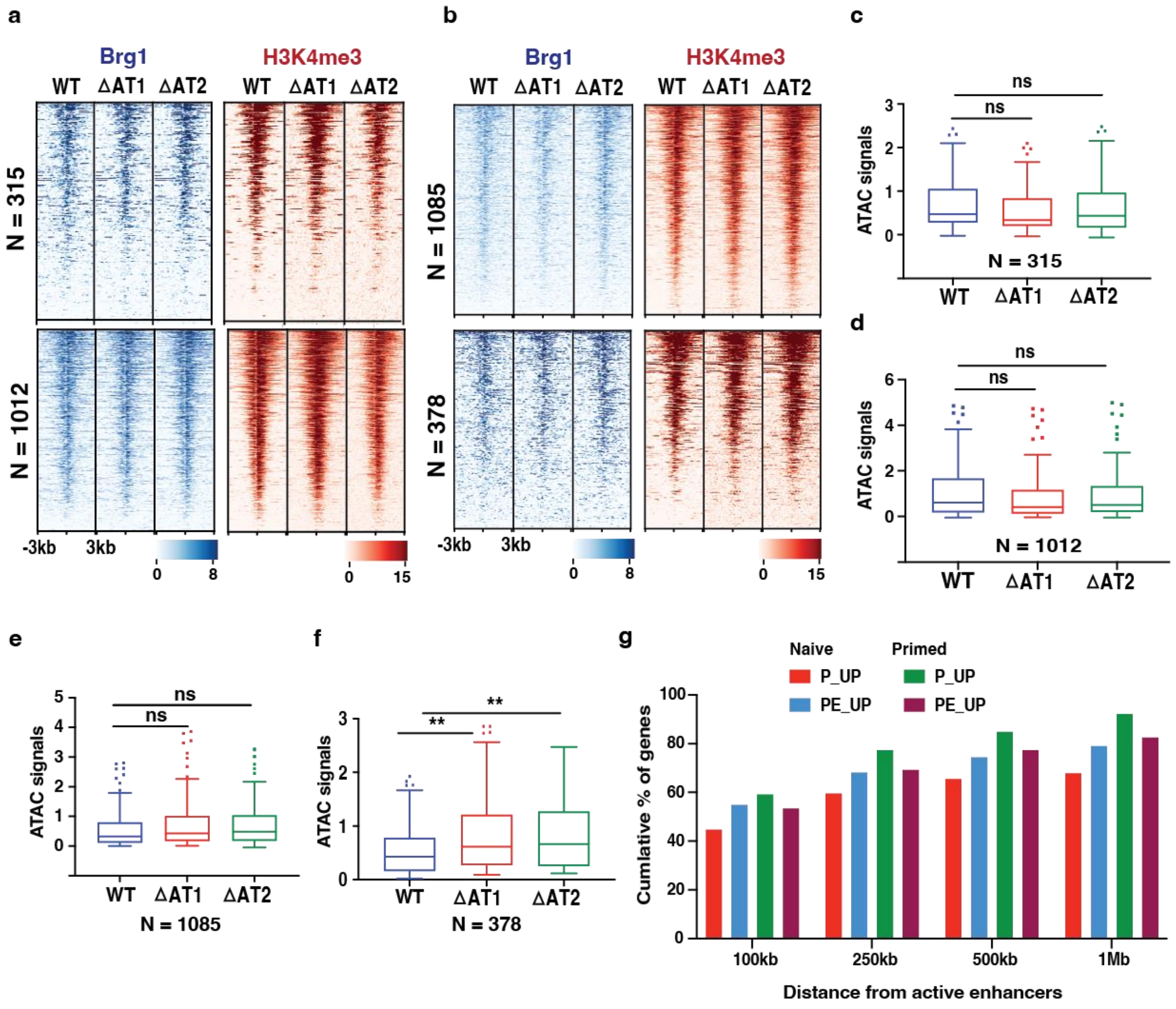

Supplementary Fig. 8: Deletion of AT-hook does not affect Brg1 localization and accessibility at promoter regions. (**a-b)** Heatmaps show localization of Brg1 (blue) and active histone mark H3K4m3 (red) in WT and AT-hook deletion clones at the promoter regions of differential genes that are being shared between dAT1 and dAT2 clones. ChIP-signals are sorted based on WT Brg1. (**c-f)** Box plots showing average ATAC-seq signals in WT and dAT mutants at the promoter regions of genes. N represents the number of genes in each group. *p<0.05; ns: not significant;

(unpaired student's t-test). The center line is the median and the box represents the group comprising the 25th to 75th percentiles. The minimum is the lowest score, excluding outliers (shown at the bottom of the lower whisker) and the maximum is the highest score, excluding outliers (shown at the top of the upper whisker). **(g)** Bar graph showing the percentage of differential-regulated genes (DE-genes) are located near the state-specific ATAC-seq peaks showing enhancer marks. Source data are provided as a Source Data File.

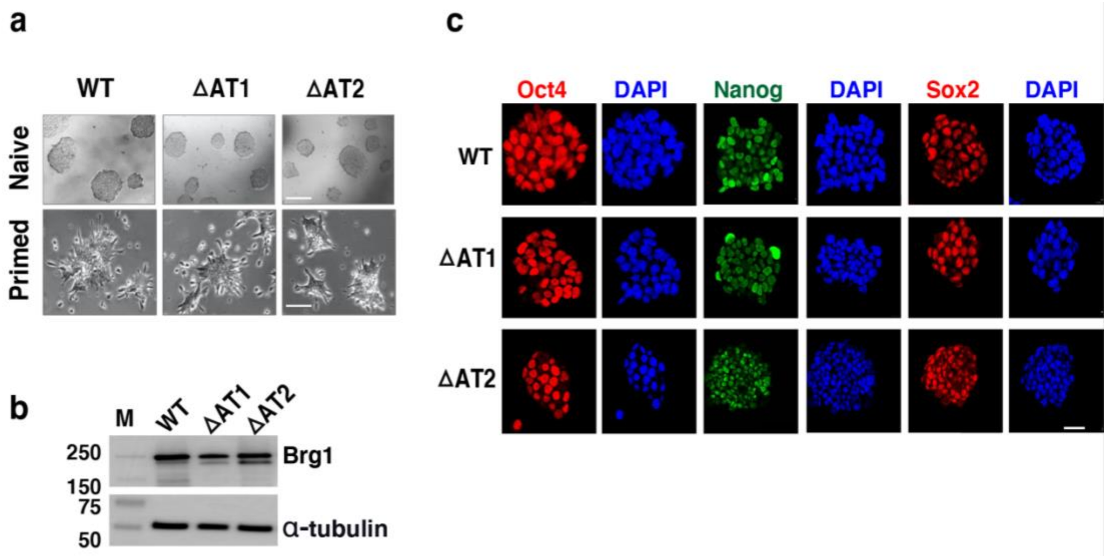

Supplementary Fig. 9: Brg1 AT-hook is dispensable for maintaining pluripotency phenotype. (**a**) Morphology of wildtype (WT) and AT-hook deletion mutants (dAT1 and dAT2) mESCs grown in naïve [(LIF/2i media supplemented with leukemia inhibitory factor (LIF), MAP/ERK kinase inhibitor (MEKi), glycogen synthase kinase 3 beta inhibitor (GSK3βi)] and primed [(media supplemented activin A, fibroblast growth factor 2 (FGF2)] conditions. (**b**) Smarca4/Brg1 expression in WT and AT-hook deletion mutants in naïve state is determined by immunoblotting; α-tubulin as a loading control. The standard protein markers are in lane M along with their molecular weight in kDa. **(c)** Immunofluorescence images showing expression and localization of the pluripotency markers Oct4, Nanog, and Sox2 in WT and AT-hook deleted mutant mESCs in naïve cells (scale bar, 20 micron). The images shown are represent of three independent replicates performed for (a-c). Source data are provided as a Source Data File.

Strain	Genotype
scSG001	MATa, his3Δ1 leu2Δ0 met15Δ0 ura3Δ0 SNF2-2xFLAG::LEU2
scSG002	MATa, his3Δ1 leu2Δ0 met15Δ0 ura3Δ0 snf2-N147S-R993H-K1279R-R1385K-S1689T-A1701T-ΔAT-2xFLAG::LEU2
scSG003	MATa, his3Δ1 leu2Δ0 met15Δ0 ura3Δ0 SNF2::LEU2
scSG004	MATa, his3Δ1 leu2Δ0 met15Δ0 ura3Δ0 snf2-ΔAT::LEU2

scSG005	MATa, his3Δ1 leu2Δ0 met15Δ0 ura3Δ0 snf2-K798A::LEU2
scSG006	MATa, his3Δ1 leu2Δ0 met15Δ0 ura3Δ0 snf2-ΔSnAC::LEU2
scSG007	MATa, his3Δ1 leu2Δ0 met15Δ0 ura3Δ0 snf2Δ::LEU2

Table 1. List of yeast strains used in this study along with their genotypes

**Name**	**primers (5'-3')**
Snf2 ΔAT hook F	tctaatcgcgactttctgctattttcacgactttcgattaattatctgccatgaacataccacagcgtcaatttagcaacgaa
Snf2 ΔAT hook r1	taatatcaataggactcgttgccgcagcgggcgtgcggacataatcaagtgcctttctctttttagacaagaaatcatc
Snf2 ΔAT hook f2	cggatttggcaatgaatgacgatgatttcttgtctaaaaagagaaaggcacttgattatgtccgcacgcccgctgcggca
Snf2 ΔAT hook R	tgtttgtctacgtataaacgaataagtacttatattgctttaggaaggtatgacgtCTAagaaaccattattatcatgac
Snf2 ΔAT hook F	tctaatcgcgactttctgctattttcacgactttcgattaattatctgccATGaacataccacagcgtcaatttagcaacgaa
Snf2 ΔAT hook 1 r1	ccagtaactggacttgattcaagcgctggtggttcagaattttcagattgcctttctctttttagacaagaaatcatc
Snf2 ΔAT hook 1 f2	cggatttggcaatgaatgacgatgatttcttgtctaaaaagagaaaggcaggatctgaaaattctgaaccaccagcgctt
Snf2 ΔAT hook R	tgtttgtctacgtataaacgaataagtacttatattgctttaggaaggtatgacgtCTAagaaaccattattatcatgac
Cdh2_Fwd	CAGGGTGGACGTCATTGTAG
Cdh2_Rev	AGGGTCTCCACCACTGATTC
Otx2_Fwd	CTTCATGAGGGAAGAGGTGG
Otx2_Rev	GGCCTCACTTTGTTCTGACC
Sox1_Fwd	CCTCGGATCTCTGGTCAAGT
Sox1_Rev	GCAGGTACATGCTGATCATCTC
Pax6_Fwd	AGTGAATGGGCGGAGTTATG
Pax6_Rev	ACTTGGACGGGAACTGACAC
Nestin_Fwd	CCCTGAAGTCGAGGAGCTG
Nestin_Rev	CTGCTGCACCTCTAAGCGA
Fgf5_Fwd	GCGATCCACAGAACTGAAAA
Fgf5_Rev	ACTGCTTGAACCTGGGTAGG
Gsc_Fwd	GCCACCGTACCATCTTCAGC
Gsc_Rev	TACGTCGGGATACTGGTTCTG
Brach_Fwd	CTGGGAGCTCAGTTCTTTCG
Brach_Rev	CCCCTTCATACATCGGAGAA
FoxA2_Fwd	GAGCAGCAACATCACCACAG
FoxA2_Rev	CGTAGGCCTTGAGGTCCAT
Gata6_Fwd	CAAAAGCTTGCTCCGGTAAC

Gata6_Rev	TGAGGTGGTCGCTTGTGTAG
Gata4_Fwd	TCTCACTATGGGCACAGCAG
Gata4_Rev	GCGATGTCTGAGTGACAGGA
Sox17_Fwd	GCTTCTCTGCCAAGGTCAAC
Sox17_Rev	CTCGGGGATGTAAAGGTGAA
Gapdh_Fwd_set3	TGACCACAGTCCATGCCATC
Gapdh_Rev_set3	GACGGACACATTGGGGGTAG

Table 2. List of primers used for yeast and mouse embryonic stem cells. The first 8 primers were used to delete the two AT-hook in yeast Snf2. The remaining primers are used for RT-PCR to monitor the expression of marker genes for the 3 basic mouse cell types (ectoderm, endoderm and mesoderm) in early development.
