# Supplementary material for: The AT-hook is an evolutionarily conserved auto-regulatory domain of SWI/SNF required for cell lineage priming
Source: Nat Commun. 2023 Aug 4;14:4682. doi: 10.1038/s41467-023-40386-8 (PMC10403523; doi:10.1038/s41467-023-40386-8)
Supplement: Supplementary file 3 — Reporting Summary [file 41467_2023_40386_MOESM3_ESM.pdf]

nature portfolio

Corresponding author(s): Blaine Bartholomew

Last updated by author(s): June 13, 2023

Reporting Summary

Nature Portfolio wishes to improve the reproducibility of the work that we publish. This form provides structure for consistency and transparency in reporting. For further information on Nature Portfolio policies, see our Editorial Policies and the Editorial Policy Checklist.

Statistics

For all statistical analyses, confirm that the following items are present in the figure legend, table legend, main text, or Methods section.

n/a	Confirmed	
☐	☒	The exact sample size (*n*) for each experimental group/condition, given as a discrete number and unit of measurement
☐	☒	A statement on whether measurements were taken from distinct samples or whether the same sample was measured repeatedly
☐	☒	The statistical test(s) used AND whether they are one- or two-sided<br>*Only common tests should be described solely by name; describe more complex techniques in the Methods section.*
☒	☐	A description of all covariates tested
☐	☒	A description of any assumptions or corrections, such as tests of normality and adjustment for multiple comparisons
☐	☒	A full description of the statistical parameters including central tendency (e.g. means) or other basic estimates (e.g. regression coefficient) AND variation (e.g. standard deviation) or associated estimates of uncertainty (e.g. confidence intervals)
☐	☒	For null hypothesis testing, the test statistic (e.g. *F*, *t*, *r*) with confidence intervals, effect sizes, degrees of freedom and *P* value noted<br>*Give P values as exact values whenever suitable.*
☒	☐	For Bayesian analysis, information on the choice of priors and Markov chain Monte Carlo settings
☒	☐	For hierarchical and complex designs, identification of the appropriate level for tests and full reporting of outcomes
☒	☐	Estimates of effect sizes (e.g. Cohen's *d*, Pearson's *r*), indicating how they were calculated

Our web collection on statistics for biologists contains articles on many of the points above.

Software and code

Policy information about availability of computer code

Data collection	Details are mentioned in the materials and method section
Data analysis	Details are mentioned in the materials and method section

For manuscripts utilizing custom algorithms or software that are central to the research but not yet described in published literature, software must be made available to editors and reviewers. We strongly encourage code deposition in a community repository (e.g. GitHub). See the Nature Portfolio guidelines for submitting code & software for further information.

Data

Policy information about availability of data

All manuscripts must include a data availability statement. This statement should provide the following information, where applicable:

- Accession codes, unique identifiers, or web links for publicly available datasets
- A description of any restrictions on data availability
- For clinical datasets or third party data, please ensure that the statement adheres to our policy

All of this is provided in the manuscript

Research involving human participants, their data, or biological material

Policy information about studies with human participants or human data. See also policy information about sex, gender (identity/presentation), and sexual orientation and race, ethnicity and racism.

Reporting on sex and gender	n/a
Reporting on race, ethnicity, or other socially relevant groupings	n/a
Population characteristics	n/a
Recruitment	n/a
Ethics oversight	n/a

Note that full information on the approval of the study protocol must also be provided in the manuscript.

Field-specific reporting

Please select the one below that is the best fit for your research. If you are not sure, read the appropriate sections before making your selection.

☒ Life sciences ☐ Behavioural & social sciences ☐ Ecological, evolutionary & environmental sciences

For a reference copy of the document with all sections, see nature.com/documents/nr-reporting-summary-flat.pdf

Life sciences study design

All studies must disclose on these points even when the disclosure is negative.

Sample size	Sample sizes based on several factors, including the variability of the data, the purpose of the experiment, the available resources, and previous research in the field.
Data exclusions	No data was excluded
Replication	All in vitro data from at least three biological replications are presented as mean ± SD. All genomics data presented here are from at least two biological replicates. . We confirm that all attempts at replication were successful.
Randomization	N/A
Blinding	N/A

Reporting for specific materials, systems and methods

We require information from authors about some types of materials, experimental systems and methods used in many studies. Here, indicate whether each material, system or method listed is relevant to your study. If you are not sure if a list item applies to your research, read the appropriate section before selecting a response.

Materials & experimental systems

n/a	Involved in the study	
☐	☒	Antibodies
☐	☒	Eukaryotic cell lines
☒	☐	Palaeontology and archaeology
☒	☐	Animals and other organisms
☒	☐	Clinical data
☒	☐	Dual use research of concern
☒	☐	Plants

Methods

n/a	Involved in the study	
☐	☒	ChIP-seq
☒	☐	Flow cytometry
☒	☐	MRI-based neuroimaging

Antibodies

Antibodies used	Antibody details are mentioned in the materials and method section; Flowing are the details of the antibodies - anti-Flag (at. No. F1804-50UG; sigma); anti-Oct4 (abcam; ab107156; 1:250 dilution), anti-Sox2 (abcam; ab107156;1:500 dilution), anti-Nanog (abcam; ab107156; 1:200 dilution), anti-Brg1/Smarca4 (abcam; ab110641; 1:1000 dilution), anti-Sox1 (CST; 4194S; 1:200

dilution), anti-Gata4 (scbt; sc-25310; 1:200 dilution), anti-HA (Invitrogen; Cat #26183; 1:1000 dilution), anti-Tubulin (ThermoFisher; A11126; 1:1000 dilution), anti-Gapdh (CST; 2118; 1:1000 dilution), anti-H3K27ac(abcam; ab4729; ChIPgrade), anti-H3K4me1(abcam; ab8895; ChIP grade), anti-H3K4me3(abcam; ab8580; ChIP grade).

Validation

All the antibodies used in the study were previously validated;
For Immunofluorescence antibodies (ab107156) following link is showing the references as presented on manufacturer's website - https://www.abcam.com/products/panels/embryonic-stem-cell-marker-panel-mouse-oct4-nanog-sox2-ssea1-ab107156.html

For Brg1/Smarca4 antibody (ab110641) -following is the link
https://www.abcam.com/products/primary-antibodies/brg1-antibody-epncir111a-ab110641.html?productwalltab=abreviews&productWallTab=Abreviews

anti-HA (Invitrogen) -https://www.thermofisher.com/antibody/product/HA-Tag-Antibody-clone-2-2-2-14-Monoclonal/26183

anti-Sox1 (CST) - https://www.cellsignal.com/products/primary-antibodies/sox1-antibody/4194

anti-Gata4 (scbt) - https://www.scbt.com/p/gata-4-antibody-g-4?gclid=CjwKCAjw-IWkBhBTEiwA2exyO_O9JZOe4OjFuq1uPVNerDpcMiv_Bei7mMrXRNbwCT4Pnn0yhE0WmxoCkQYQAvD_BwE

anti-H3K4me3 - https://www.abcam.com/products/primary-antibodies/histone-h3-tri-methyl-k4-antibody-chip-grade-ab8580.html

anti-H3K27ac - https://www.abcam.com/products/primary-antibodies/histone-h3-acetyl-k27-antibody-chip-grade-ab4729.html

anti-H3K4me1 - https://www.abcam.com/products/primary-antibodies/histone-h3-mono-methyl-k4-antibody-chip-grade-ab8895.html

anti-Gapdh - https://www.cellsignal.com/products/primary-antibodies/gapdh-14c10-rabbit-mab/2118

anti-tubulin - https://www.thermofisher.com/antibody/product/alpha-Tubulin-Antibody-clone-236-10501-Monoclonal/A11126

Eukaryotic cell lines

Policy information about cell lines and Sex and Gender in Research

Cell line source(s)	Mouse ES Cell line was procured from ATCC
Authentication	Authentication was done using cell morphology and growth properties as mentioned in ATCC guidelines
Mycoplasma contamination	Cell line was tested negative for mycoplasma contamination using MyCo Alert kit from Lonza
Commonly misidentified lines (See ICLAC register)	Cell lines used in this work was not listed as "Misidentified Cell Line" in the ICLAC database.

Plants

Seed stocks	n/a
Novel plant genotypes	n/a
Authentication	n/a

ChIP-seq

Data deposition

☒ Confirm that both raw and final processed data have been deposited in a public database such as GEO.

☐ Confirm that you have deposited or provided access to graph files (e.g. BED files) for the called peaks.

Data access links
May remain private before publication.

GSE207793

Files in database submission

Brg1_In1_WT_FBS_2i_rep1_S1_L001_R1_001.fastq.gz Brg1_In1_WT_FBS_2i_rep1_S1_L002_R1_001.fastq.gz
Brg1_In1_WT_FBS_2i_rep1_S1_L003_R1_001.fastq.gz Brg1_In1_WT_FBS_2i_rep1_S1_L004_R1_001.fastq.gz
Brg1_In1_WT_FBS_2i_rep1_S1_L001_R2_001.fastq.gz Brg1_In1_WT_FBS_2i_rep1_S1_L002_R2_001.fastq.gz
Brg1_In1_WT_FBS_2i_rep1_S1_L003_R2_001.fastq.gz Brg1_In1_WT_FBS_2i_rep1_S1_L004_R2_001.fastq.gz
Brg1_In1_WT_FBS_2i_rep2_S1_L001_R1_001.fastq.gz Brg1_In1_WT_FBS_2i_rep2_S1_L002_R1_001.fastq.gz
Brg1_In1_WT_FBS_2i_rep2_S1_L003_R1_001.fastq.gz Brg1_In1_WT_FBS_2i_rep2_S1_L004_R1_001.fastq.gz
Brg1_In1_WT_FBS_2i_rep2_S1_L001_R2_001.fastq.gz Brg1_In1_WT_FBS_2i_rep2_S1_L002_R2_001.fastq.gz
Brg1_In1_WT_FBS_2i_rep2_S1_L003_R2_001.fastq.gz Brg1_In1_WT_FBS_2i_rep2_S1_L004_R2_001.fastq.gz

Brg1_In4_WT_CDM_rep1_S2_L001_R1_001.fastq.gz Brg1_In4_WT_CDM_rep1_S2_L002_R1_001.fastq.gz
Brg1_In4_WT_CDM_rep1_S2_L003_R1_001.fastq.gz Brg1_In4_WT_CDM_rep1_S2_L004_R1_001.fastq.gz
Brg1_In4_WT_CDM_rep1_S2_L001_R2_001.fastq.gz Brg1_In4_WT_CDM_rep1_S2_L002_R2_001.fastq.gz
Brg1_In4_WT_CDM_rep1_S2_L003_R2_001.fastq.gz Brg1_In4_WT_CDM_rep1_S2_L004_R2_001.fastq.gz
Brg1_In4_WT_CDM_rep2_S2_L001_R1_001.fastq.gz Brg1_In4_WT_CDM_rep2_S2_L002_R1_001.fastq.gz
Brg1_In4_WT_CDM_rep2_S2_L003_R1_001.fastq.gz Brg1_In4_WT_CDM_rep2_S2_L004_R1_001.fastq.gz
Brg1_In4_WT_CDM_rep2_S2_L001_R2_001.fastq.gz Brg1_In4_WT_CDM_rep2_S2_L002_R2_001.fastq.gz
Brg1_In4_WT_CDM_rep2_S2_L003_R2_001.fastq.gz Brg1_In4_WT_CDM_rep2_S2_L004_R2_001.fastq.gz
Brg1_In8_A2_FBS_2i_rep1_S5_L001_R1_001.fastq.gz Brg1_In8_A2_FBS_2i_rep1_S5_L002_R1_001.fastq.gz
Brg1_In8_A2_FBS_2i_rep1_S5_L003_R1_001.fastq.gz Brg1_In8_A2_FBS_2i_rep1_S5_L004_R1_001.fastq.gz
Brg1_In8_A2_FBS_2i_rep1_S5_L001_R2_001.fastq.gz Brg1_In8_A2_FBS_2i_rep1_S5_L002_R2_001.fastq.gz
Brg1_In8_A2_FBS_2i_rep1_S5_L003_R2_001.fastq.gz Brg1_In8_A2_FBS_2i_rep1_S5_L004_R2_001.fastq.gz
Brg1_In8_A2_FBS_2i_rep2_S5_L001_R1_001.fastq.gz Brg1_In8_A2_FBS_2i_rep2_S5_L002_R1_001.fastq.gz
Brg1_In8_A2_FBS_2i_rep2_S5_L003_R1_001.fastq.gz Brg1_In8_A2_FBS_2i_rep2_S5_L004_R1_001.fastq.gz
Brg1_In8_A2_FBS_2i_rep2_S5_L001_R2_001.fastq.gz Brg1_In8_A2_FBS_2i_rep2_S5_L002_R2_001.fastq.gz
Brg1_In8_A2_FBS_2i_rep2_S5_L003_R2_001.fastq.gz Brg1_In8_A2_FBS_2i_rep2_S5_L004_R2_001.fastq.gz
Brg1_In10_A2_CDM_rep1_S3_L001_R1_001.fastq.gz Brg1_In10_A2_CDM_rep1_S3_L002_R1_001.fastq.gz
Brg1_In10_A2_CDM_rep1_S3_L003_R1_001.fastq.gz Brg1_In10_A2_CDM_rep1_S3_L004_R1_001.fastq.gz
Brg1_In10_A2_CDM_rep1_S3_L001_R2_001.fastq.gz Brg1_In10_A2_CDM_rep1_S3_L002_R2_001.fastq.gz
Brg1_In10_A2_CDM_rep1_S3_L003_R2_001.fastq.gz Brg1_In10_A2_CDM_rep1_S3_L004_R2_001.fastq.gz
Brg1_In10_A2_CDM_rep2_S3_L001_R1_001.fastq.gz Brg1_In10_A2_CDM_rep2_S3_L002_R1_001.fastq.gz
Brg1_In10_A2_CDM_rep2_S3_L003_R1_001.fastq.gz Brg1_In10_A2_CDM_rep2_S3_L004_R1_001.fastq.gz
Brg1_In10_A2_CDM_rep2_S3_L001_R2_001.fastq.gz Brg1_In10_A2_CDM_rep2_S3_L002_R2_001.fastq.gz
Brg1_In10_A2_CDM_rep2_S3_L003_R2_001.fastq.gz Brg1_In10_A2_CDM_rep2_S3_L004_R2_001.fastq.gz
Brg1_In5_D8_FBS_2i_rep1_S3_L001_R1_001.fastq.gz Brg1_In5_D8_FBS_2i_rep1_S3_L002_R1_001.fastq.gz
Brg1_In5_D8_FBS_2i_rep1_S3_L003_R1_001.fastq.gz Brg1_In5_D8_FBS_2i_rep1_S3_L004_R1_001.fastq.gz
Brg1_In5_D8_FBS_2i_rep1_S3_L001_R2_001.fastq.gz Brg1_In5_D8_FBS_2i_rep1_S3_L002_R2_001.fastq.gz
Brg1_In5_D8_FBS_2i_rep1_S3_L003_R2_001.fastq.gz Brg1_In5_D8_FBS_2i_rep1_S3_L004_R2_001.fastq.gz
Brg1_In5_D8_FBS_2i_rep2_S3_L001_R1_001.fastq.gz Brg1_In5_D8_FBS_2i_rep2_S3_L002_R1_001.fastq.gz
Brg1_In5_D8_FBS_2i_rep2_S3_L003_R1_001.fastq.gz Brg1_In5_D8_FBS_2i_rep2_S3_L004_R1_001.fastq.gz
Brg1_In5_D8_FBS_2i_rep2_S3_L001_R2_001.fastq.gz Brg1_In5_D8_FBS_2i_rep2_S3_L002_R2_001.fastq.gz
Brg1_In5_D8_FBS_2i_rep2_S3_L003_R2_001.fastq.gz Brg1_In5_D8_FBS_2i_rep2_S3_L004_R2_001.fastq.gz
Brg1_In7_D8_CDM_rep1_S2_L001_R1_001.fastq.gz Brg1_In7_D8_CDM_rep1_S2_L002_R1_001.fastq.gz
Brg1_In7_D8_CDM_rep1_S2_L003_R1_001.fastq.gz Brg1_In7_D8_CDM_rep1_S2_L004_R1_001.fastq.gz
Brg1_In7_D8_CDM_rep1_S2_L001_R2_001.fastq.gz Brg1_In7_D8_CDM_rep1_S2_L002_R2_001.fastq.gz
Brg1_In7_D8_CDM_rep1_S2_L003_R2_001.fastq.gz Brg1_In7_D8_CDM_rep1_S2_L004_R2_001.fastq.gz
Brg1_In7_D8_CDM_rep2_S2_L001_R1_001.fastq.gz Brg1_In7_D8_CDM_rep2_S2_L002_R1_001.fastq.gz
Brg1_In7_D8_CDM_rep2_S2_L003_R1_001.fastq.gz Brg1_In7_D8_CDM_rep2_S2_L004_R1_001.fastq.gz
Brg1_In7_D8_CDM_rep2_S2_L001_R2_001.fastq.gz Brg1_In7_D8_CDM_rep2_S2_L002_R2_001.fastq.gz
Brg1_In7_D8_CDM_rep2_S2_L003_R2_001.fastq.gz Brg1_In7_D8_CDM_rep2_S2_L004_R2_001.fastq.gz
H3K4me1_Sample3_WT_FBS_2i_rep1_S1_L001_R1_001.fastq.gz
H3K4me1_Sample3_WT_FBS_2i_rep1_S1_L002_R1_001.fastq.gz
H3K4me1_Sample3_WT_FBS_2i_rep1_S1_L003_R1_001.fastq.gz
H3K4me1_Sample3_WT_FBS_2i_rep1_S1_L004_R1_001.fastq.gz
H3K4me1_Sample3_WT_FBS_2i_rep1_S1_L001_R2_001.fastq.gz
H3K4me1_Sample3_WT_FBS_2i_rep1_S1_L002_R2_001.fastq.gz
H3K4me1_Sample3_WT_FBS_2i_rep1_S1_L003_R2_001.fastq.gz
H3K4me1_Sample3_WT_FBS_2i_rep1_S1_L004_R2_001.fastq.gz
H3K4me1_Sample4_WT_FBS_2i_rep2_S2_L001_R1_001.fastq.gz
H3K4me1_Sample4_WT_FBS_2i_rep2_S2_L002_R1_001.fastq.gz
H3K4me1_Sample4_WT_FBS_2i_rep2_S2_L003_R1_001.fastq.gz
H3K4me1_Sample4_WT_FBS_2i_rep2_S2_L004_R1_001.fastq.gz
H3K4me1_Sample4_WT_FBS_2i_rep2_S2_L001_R2_001.fastq.gz
H3K4me1_Sample4_WT_FBS_2i_rep2_S2_L002_R2_001.fastq.gz
H3K4me1_Sample4_WT_FBS_2i_rep2_S2_L003_R2_001.fastq.gz
H3K4me1_Sample4_WT_FBS_2i_rep2_S2_L004_R2_001.fastq.gz
H3K4me1_Sample51_WT_CDM_rep1_S5_L001_R1_001.fastq.gz
H3K4me1_Sample51_WT_CDM_rep1_S5_L002_R1_001.fastq.gz
H3K4me1_Sample51_WT_CDM_rep1_S5_L003_R1_001.fastq.gz
H3K4me1_Sample51_WT_CDM_rep1_S5_L004_R1_001.fastq.gz
H3K4me1_Sample51_WT_CDM_rep1_S5_L001_R2_001.fastq.gz
H3K4me1_Sample51_WT_CDM_rep1_S5_L002_R2_001.fastq.gz
H3K4me1_Sample51_WT_CDM_rep1_S5_L003_R2_001.fastq.gz
H3K4me1_Sample51_WT_CDM_rep1_S5_L004_R2_001.fastq.gz
H3K4me1_Sample52_WT_CDM_rep2_S6_L001_R1_001.fastq.gz
H3K4me1_Sample52_WT_CDM_rep2_S6_L002_R1_001.fastq.gz
H3K4me1_Sample52_WT_CDM_rep2_S6_L003_R1_001.fastq.gz
H3K4me1_Sample52_WT_CDM_rep2_S6_L004_R1_001.fastq.gz
H3K4me1_Sample52_WT_CDM_rep2_S6_L001_R2_001.fastq.gz
H3K4me1_Sample52_WT_CDM_rep2_S6_L002_R2_001.fastq.gz
H3K4me1_Sample52_WT_CDM_rep2_S6_L003_R2_001.fastq.gz
H3K4me1_Sample52_WT_CDM_rep2_S6_L004_R2_001.fastq.gz
H3K4me3_Sample1_WT_FBS_2i_rep1_S1_L001_R1_001.fastq.gz
H3K4me3_Sample1_WT_FBS_2i_rep1_S1_L002_R1_001.fastq.gz
H3K4me3_Sample1_WT_FBS_2i_rep1_S1_L003_R1_001.fastq.gz
H3K4me3_Sample1_WT_FBS_2i_rep1_S1_L004_R1_001.fastq.gz

H3K4me3_Sample1_WT_FBS_2i_rep1_S1_L001_R2_001.fastq.gz
H3K4me3_Sample1_WT_FBS_2i_rep1_S1_L002_R2_001.fastq.gz
H3K4me3_Sample1_WT_FBS_2i_rep1_S1_L003_R2_001.fastq.gz
H3K4me3_Sample1_WT_FBS_2i_rep1_S1_L004_R2_001.fastq.gz
H3K4me3_Sample2_WT_FBS_2i_rep2_S2_L001_R1_001.fastq.gz
H3K4me3_Sample2_WT_FBS_2i_rep2_S2_L002_R1_001.fastq.gz
H3K4me3_Sample2_WT_FBS_2i_rep2_S2_L003_R1_001.fastq.gz
H3K4me3_Sample2_WT_FBS_2i_rep2_S2_L004_R1_001.fastq.gz
H3K4me3_Sample2_WT_FBS_2i_rep2_S2_L001_R2_001.fastq.gz
H3K4me3_Sample2_WT_FBS_2i_rep2_S2_L002_R2_001.fastq.gz
H3K4me3_Sample2_WT_FBS_2i_rep2_S2_L003_R2_001.fastq.gz
H3K4me3_Sample2_WT_FBS_2i_rep2_S2_L004_R2_001.fastq.gz
H3K4me3_Sample49_WT_CDM_rep1_S5_L001_R1_001.fastq.gz
H3K4me3_Sample49_WT_CDM_rep1_S5_L002_R1_001.fastq.gz
H3K4me3_Sample49_WT_CDM_rep1_S5_L003_R1_001.fastq.gz
H3K4me3_Sample49_WT_CDM_rep1_S5_L004_R1_001.fastq.gz
H3K4me3_Sample49_WT_CDM_rep1_S5_L001_R2_001.fastq.gz
H3K4me3_Sample49_WT_CDM_rep1_S5_L002_R2_001.fastq.gz
H3K4me3_Sample49_WT_CDM_rep1_S5_L003_R2_001.fastq.gz
H3K4me3_Sample49_WT_CDM_rep1_S5_L004_R2_001.fastq.gz
H3K4me3_Sample50_WT_CDM_rep2_S6_L001_R1_001.fastq.gz
H3K4me3_Sample50_WT_CDM_rep2_S6_L002_R1_001.fastq.gz
H3K4me3_Sample50_WT_CDM_rep2_S6_L003_R1_001.fastq.gz
H3K4me3_Sample50_WT_CDM_rep2_S6_L004_R1_001.fastq.gz
H3K4me3_Sample50_WT_CDM_rep2_S6_L001_R2_001.fastq.gz
H3K4me3_Sample50_WT_CDM_rep2_S6_L002_R2_001.fastq.gz
H3K4me3_Sample50_WT_CDM_rep2_S6_L003_R2_001.fastq.gz
H3K4me3_Sample50_WT_CDM_rep2_S6_L004_R2_001.fastq.gz
H3K27ac_Sample7_WT_FBS_2i_rep1_S1_L001_R1_001.fastq.gz
H3K27ac_Sample7_WT_FBS_2i_rep1_S1_L002_R1_001.fastq.gz
H3K27ac_Sample7_WT_FBS_2i_rep1_S1_L003_R1_001.fastq.gz
H3K27ac_Sample7_WT_FBS_2i_rep1_S1_L004_R1_001.fastq.gz
H3K27ac_Sample7_WT_FBS_2i_rep1_S1_L001_R2_001.fastq.gz
H3K27ac_Sample7_WT_FBS_2i_rep1_S1_L002_R2_001.fastq.gz
H3K27ac_Sample7_WT_FBS_2i_rep1_S1_L003_R2_001.fastq.gz
H3K27ac_Sample7_WT_FBS_2i_rep1_S1_L004_R2_001.fastq.gz
H3K27ac_Sample8_WT_FBS_2i_rep2_S2_L001_R1_001.fastq.gz
H3K27ac_Sample8_WT_FBS_2i_rep2_S2_L002_R1_001.fastq.gz
H3K27ac_Sample8_WT_FBS_2i_rep2_S2_L003_R1_001.fastq.gz
H3K27ac_Sample8_WT_FBS_2i_rep2_S2_L004_R1_001.fastq.gz
H3K27ac_Sample8_WT_FBS_2i_rep2_S2_L001_R2_001.fastq.gz
H3K27ac_Sample8_WT_FBS_2i_rep2_S2_L002_R2_001.fastq.gz
H3K27ac_Sample8_WT_FBS_2i_rep2_S2_L003_R2_001.fastq.gz
H3K27ac_Sample8_WT_FBS_2i_rep2_S2_L004_R2_001.fastq.gz
H3K27ac_Sample55_WT_CDM_rep1_S5_L001_R1_001.fastq.gz
H3K27ac_Sample55_WT_CDM_rep1_S5_L002_R1_001.fastq.gz
H3K27ac_Sample55_WT_CDM_rep1_S5_L003_R1_001.fastq.gz
H3K27ac_Sample55_WT_CDM_rep1_S5_L004_R1_001.fastq.gz
H3K27ac_Sample55_WT_CDM_rep1_S5_L001_R2_001.fastq.gz
H3K27ac_Sample55_WT_CDM_rep1_S5_L002_R2_001.fastq.gz
H3K27ac_Sample55_WT_CDM_rep1_S5_L003_R2_001.fastq.gz
H3K27ac_Sample55_WT_CDM_rep1_S5_L004_R2_001.fastq.gz
H3K27ac_Sample56_WT_CDM_rep2_S6_L001_R1_001.fastq.gz
H3K27ac_Sample56_WT_CDM_rep2_S6_L002_R1_001.fastq.gz
H3K27ac_Sample56_WT_CDM_rep2_S6_L003_R1_001.fastq.gz
H3K27ac_Sample56_WT_CDM_rep2_S6_L004_R1_001.fastq.gz
H3K27ac_Sample56_WT_CDM_rep2_S6_L001_R2_001.fastq.gz
H3K27ac_Sample56_WT_CDM_rep2_S6_L002_R2_001.fastq.gz
H3K27ac_Sample56_WT_CDM_rep2_S6_L003_R2_001.fastq.gz
H3K27ac_Sample56_WT_CDM_rep2_S6_L004_R2_001.fastq.gz
Input1_WT_FBS_2i_S1_L001_R1_001.fastq.gz Input1_WT_FBS_2i_S1_L002_R1_001.fastq.gz
Input1_WT_FBS_2i_S1_L003_R1_001.fastq.gz Input1_WT_FBS_2i_S1_L004_R1_001.fastq.gz
Input1_WT_FBS_2i_S1_L001_R2_001.fastq.gz Input1_WT_FBS_2i_S1_L002_R2_001.fastq.gz
Input1_WT_FBS_2i_S1_L003_R2_001.fastq.gz Input1_WT_FBS_2i_S1_L004_R2_001.fastq.gz
Input4_WT_CDM_S3_L001_R1_001.fastq.gz Input4_WT_CDM_S3_L002_R1_001.fastq.gz
Input4_WT_CDM_S3_L003_R1_001.fastq.gz Input4_WT_CDM_S3_L004_R1_001.fastq.gz
Input4_WT_CDM_S3_L001_R2_001.fastq.gz Input4_WT_CDM_S3_L002_R2_001.fastq.gz
Input4_WT_CDM_S3_L003_R2_001.fastq.gz Input4_WT_CDM_S3_L004_R2_001.fastq.gz

Processed data file name -

WT_Brg1_2i_merge.subtract.bw
WT_Brg1_2i_merge.subtract.bw
WT_Brg1_CDM_merge.subtract.bw
WT_Brg1_CDM_merge.subtract.bw
A2_Brg1_2i_merge.subtract.bw

A2_Brg1_2i_merge.subtract.bw
A2_Brg1_CDM_merge.subtract.bw
A2_Brg1_CDM_merge.subtract.bw
D8_Brg1_2i_merge.subtract.bw
D8_Brg1_2i_merge.subtract.bw
D8_Brg1_CDM_merge.subtract.bw
D8_Brg1_CDM_merge.subtract.bw
WT_H3K4me1_WT_2i_merge.subtract.bw
WT_H3K4me1_WT_2i_merge.subtract.bw
WT_H3K4me1_WT_CDM_merge.subtract.bw
WT_H3K4me1_WT_CDM_merge.subtract.bw
A2_H3K4me1_2i_merge.bw
A2_H3K4me1_2i_merge.bw
A2_H3K4me1_CDM_merge.bw
A2_H3K4me1_CDM_merge.bw
D8_H3K4me1_2i_merge.bw
D8_H3K4me1_2i_merge.bw
D8_H3K4me1_CDM_merge.bw
D8_H3K4me1_CDM_merge.bw
WT_H3K4me3_2i_merge.subtract.bw
WT_H3K4me3_2i_merge.subtract.bw
WT_H3K4me3_CDM_merge.subtract.bw
WT_H3K4me3_CDM_merge.subtract.bw
A2_H3K4me3_2i_merge.bw
A2_H3K4me3_2i_merge.bw
A2_H3K4me3_CDM_merge.bw
A2_H3K4me3_CDM_merge.bw
D8_H3K4me3_2i_merge.bw
D8_H3K4me3_2i_merge.bw
D8_H3K4me3_CDM_merge.bw
D8_H3K4me3_CDM_merge.bw
WT_H3K27ac_2i_merge.subtract.bw
WT_H3K27ac_2i_merge.subtract.bw
WT_H3K27ac_CDM_merge.subtract.bw
WT_H3K27ac_CDM_merge.subtract.bw
A2_H3K27ac_2i_merge.bw
A2_H3K27ac_2i_merge.bw
A2_H3K27ac_CDM_merge.bw
A2_H3K27ac_CDM_merge.bw
D8_H3K27ac_2i_merge.bw
D8_H3K27ac_2i_merge.bw
D8_H3K27ac_CDM_merge.bw
D8_H3K27ac_CDM_merge.bw
WT_Brg1_2i_merge.subtract.bw WT_H3K4me1_WT_2i_merge.subtract.bw WT_H3K4me3_2i_merge.subtract.bw
WT_H3K27ac_2i_merge.subtract.bw
WT_Brg1_CDM_merge.subtract.bw WT_H3K4me1_WT_CDM_merge.subtract.bw WT_H3K4me3_CDM_merge.subtract.bw
WT_H3K27ac_CDM_merge.subtract.bw
A2_Brg1_2i_merge.subtract.bw A2_H3K4me1_2i_merge.bw A2_H3K4me3_2i_merge.bw A2_H3K27ac_2i_merge.bw
A2_Brg1_2i_merge.subtract.bw A2_H3K4me1_2i_merge.bw A2_H3K4me3_2i_merge.bw A2_H3K27ac_2i_merge.bw
A2_Brg1_CDM_merge.subtract.bw A2_H3K4me1_CDM_merge.bw A2_H3K4me3_CDM_merge.bw
A2_H3K27ac_CDM_merge.bw
A2_Brg1_CDM_merge.subtract.bw A2_H3K4me1_CDM_merge.bw A2_H3K4me3_CDM_merge.bw
A2_H3K27ac_CDM_merge.bw
D8_Brg1_2i_merge.subtract.bw D8_H3K4me1_2i_merge.bw D8_H3K4me3_2i_merge.bw D8_H3K27ac_2i_merge.bw
D8_Brg1_2i_merge.subtract.bw D8_H3K4me1_2i_merge.bw D8_H3K4me3_2i_merge.bw D8_H3K27ac_2i_merge.bw
D8_Brg1_CDM_merge.subtract.bw D8_H3K4me1_CDM_merge.bw D8_H3K4me3_CDM_merge.bw
D8_H3K27ac_CDM_merge.bw
D8_Brg1_CDM_merge.subtract.bw D8_H3K4me1_CDM_merge.bw D8_H3K4me3_CDM_merge.bw
D8_H3K27ac_CDM_merge.bw

Genome browser session (e.g. UCSC)

N/A

Methodology

Replicates

Each experiment has two biological replicates and both replicates are strongly matched

Sequencing depth

Over 40M reads were obtained for Brg1 and Histone ChIP-seq using paired end 75bp sequencing.;More than 80% reads were uniquely mapped and 30M reads were used for downstream analysis;

Antibodies

Following are the details of the antibody -

For Brg1/Smarca4 antibody (ab110641) -following is the link
https://www.abcam.com/products/primary-antibodies/brg1-antibody-epncir111a-ab110641.html?productwalltab=abreviews&productWallTab=Abreviews

anti-H3K4me3 - https://www.abcam.com/products/primary-antibodies/histone-h3-tri-methyl-k4-antibody-chip-grade-ab8580.html

anti-H3K27ac - https://www.abcam.com/products/primary-antibodies/histone-h3-acetyl-k27-antibody-chip-grade-ab4729.html

anti-H3K4me1 - https://www.abcam.com/products/primary-antibodies/histone-h3-mono-methyl-k4-antibody-chip-grade-ab8895.html

Peak calling parameters	For histone and Brg1 ChIP, peaks were called using MACS2 with the following parameters: --broad -q 0.05 –nomodel -extsize 500
Data quality	Details are mentioned in the data analysis section under Materials and Method; Fastq files are also deposited in the database. All the ChIP-seq samples were tested for cross-correlation, multi-QC and FastQC
Software	Software details are mentioned in the method section; No custom software was used for the analysis; Briefly following are available softwares were used for data analysis - Trim Galore for trimming reads with default parameter, samtools for fastq to bam conversion, Reads were aligned to mouse mm10 using BOWTIE2 (very-sensitive method), MACS2 was used for peak calling, Deeptools for ChIP-seq heatmap analysis, bedtools, HOMER for motif analysis, ggplot from R package for meta-analysis of PRO-seq, TOBIAS for ATAC foot-printing
